# Benchmarking and Automating the Biotinylation Proteomics Workflow

**DOI:** 10.21203/rs.3.rs-4590410/v1

**Published:** 2024-07-03

**Authors:** Haorong Li, Noah Smeriglio, Jiawei Ni, Yan Wang, Shiori Sekine, Ling Hao

**Affiliations:** 1 Department of Chemistry, The George Washington University, Washington, DC, 20052, USA; 2 National Institute of Dental and Craniofacial Research, National Institutes of Health, Bethesda, MD, 20892, USA; 3 Aging Institute, Department of Cell Biology, School of Medicine, University of Pittsburgh, Pittsburgh, PA, 15219, USA

## Abstract

Protein biotinylation has been widely used in biotechnology with various labeling and enrichment strategies. However, different enrichment strategies have not been systematically evaluated due to the lack of a benchmarking model for fair comparison. Most biotinylation proteomics workflows suffer from lengthy experimental steps, non-specific bindings, limited throughput, and experimental variability. To address these challenges, we designed a two-proteome model, where biotinylated yeast proteins were spiked in unlabeled human proteins, allowing us to distinguish true enrichment from non-specific bindings. Using this benchmarking model, we compared common biotinylation proteomics methods and provided practical selection guidelines. We significantly optimized and shortened sample preparation from 3 days to 9 hours, enabling fully-automated 96-well plate sample processing. Next, we applied this optimized and automated workflow for proximity labeling to investigate the intricate interplay between mitochondria and lysosomes in living cells under both healthy state and mitochondrial damage. Our results suggested a time-dependent proteome remodeling and dynamic translocation within mitochondria and between mitochondria and lysosomes upon mitochondrial damage. This newly established benchmarking model and the fully-automated 9-hour workflow can be readily applied to the broad fields of protein biotinylation to study protein interaction and organelle dynamics.

## Introduction

Protein biotinylation has been widely used in biotechnology for affinity purification, immobilization, imaging, and drug delivery^1–3^. By chemically or enzymatically attaching biotin moiety to target proteins, researchers can enrich biotinylated proteins from complex mixtures using affinity beads to identify true biological targets. Streptavidin and neutravidin coated beads are commonly used for biotin enrichment due to their exceptional affinity for biotinylated proteins. However, the strong affinity poses challenge during elution. On-bead digestion is often used to elute biotinylated proteins but suffers from highly abundant streptavidin contamination and limited coverage of biotinylation sites^3–5^. Recent advances aimed to address these challenges with the development of protease-resistant streptavidin beads, low affinity biotin substrates, cleavable biotins, and biotin antibody^6–11^. Biotin antibody or neutravidin coated beads have also been used for peptide-level enrichment to specifically enrich biotinylated peptides^10–12^. While various enrichment strategies are available, there has not been a direct comparison among these methods because of the lack of a benchmarking model for fair comparison. Comparing these biotinylation proteomics workflow and providing practical user guidelines for method selection and experimental design are essential for advancing protein biotinylation research and applications.

A typical biotinylation proteomics sample preparation includes overnight enrichment of biotinylated proteins, extensive bead washes to reduce non-specific bindings, biotinylation elution, protein reduction and alkylation, overnight protein digestion, and peptide clean-up. This workflow typically takes three days to finish, and is prone to human errors and challenging to scale up. Recent advances in proteomics automation enabled high-throughput sample processing, less variability, and shortened hands-on time via liquid handlers or magnetic bead processor^13–17^. However, the lengthy sample preparation for biotinylation proteomics workflow with two overnight incubation steps is not feasible to fully automate in an open-air automated platform. Detergent-based buffers are often required in an automated system to ensure smooth movement of magnetic beads, which could limit the selection of downstream peptide clean-up methods and cause contaminations to liquid chromatography-mass spectrometry (LC-MS) instruments. Therefore, optimizing the biotinylation workflow is necessary before transitioning from manual processing to a fully automated pipeline.

Proximity labeling represents a major application for biotinylation proteomics, which has been widely used to define protein networks and organelle microenvironment surrounding a bait protein in living cells and organisms^18,19^. Engineered peroxidases (e.g., APEX^20^) or biotin ligases (e.g., BioID^21^, TurboID^22^) can be tagged onto the bait protein to biotinylate neighboring prey proteins within a 10 nm radius upon activation, which can then be enriched and analyzed by MS-proteomics. Proximity labeling can be a promising tool to study organelle interactions but has rarely been used to capture the precise inter-organelle membrane contact sites, partly due to the technological limitations of biotinylation proteomics mentioned above. Addressing the technical challenges of biotinylation proteomics and proximity labeling could open a new frontier to capture inter-organelle membrane contact sites and organelle dynamics with unprecedented precision, sensitivity, reproducibility, and throughput.

In this study, we first designed a two-proteome benchmarking model, where a small amount of yeast proteins was biotinylated *in vitro* and spiked in unlabeled human proteins, allowing us to mimic protein biotinylation in a biological system and distinguish true enrichment (yeast) from non-specific bindings (human). Using this two-proteome model, different biotinylation enrichment strategies were systematically compared at both the protein-level and peptide-level enrichments, regarding the binding capacity, non-specific interferences, biotinylated proteome coverage, and biotinylation site identification. The entire sample preparation workflow was extensively optimized and shortened from the traditional 3 days to just 9 hours, enabling a fully-automated 96-well plate format of sample processing. We then demonstrated the applicability of this optimized and automated 9-hour workflow in proximity labeling proteomics at different subcellular locations. We identified and quantified biotinylated proteins with precise amino acid residues from mitochondria, lysosomes, mitochondria-lysosome contact sites, and their cytosolic interactions in living cells. Our proteomics results suggested the dynamic proteome remodeling and time-dependent translocation within different compartments of mitochondria and between mitochondria and lysosomes upon mitochondrial damage.

## Results

### Benchmarking biotinylation enrichment methods using a two-proteome model

**Fig. 1:**
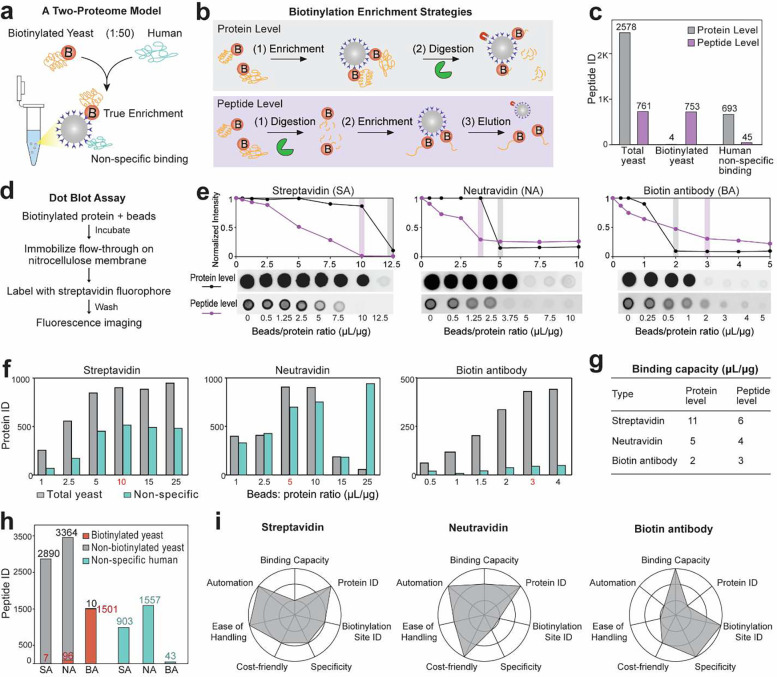
Benchmarking biotinylation enrichment methods. (a) Schematics of a two-proteome model to distinguish true enrichment (yeast) from non-specific binding proteins (human). (b) Schematic workflow of protein-level and peptide-level biotinylation enrichments. (c) Comparison of yeast and human peptide IDs between protein-level and peptide-level biotinylation enrichments using SA-coated beads. Protein ID comparisons are provided in Supplementary Fig. S1. (d) Schematics of a dot blot assay for beads titration. (e) Dot blot assay to determine binding capacity of SA, NA, and BA beads. The y-axis shows the remaining biotinylation signals in the bead-protein mixture supernatant after enrichment. Optimal ratios are highlighted. (f) Proteomics results showing identified and quantified protein IDs from SA, NA, and BA enrichments using 10 μg of biotinylated yeast proteins and different beads amount. Optimal ratios are marked in red. True enrichment of yeast proteins and non-specific human proteins are labeled in different colors. Peptide ID comparisons are provided in Supplementary Fig. S1. (g) Beads binding capacity (μL of beads/μg of input proteins) averaged from dot blot assay and proteomics results. (h) Comparison of peptide IDs from SA, NA and BA using their optimal beads/protein ratios. (i) Radar plots comparing the performances of SA, NA, and BA methods for biotinylation enrichment.

An ideal biotinylation enrichment should sufficiently capture biotinylated proteins while minimizing non-specific bindings. Here, we designed a two-proteome model, where a small amount of yeast proteins was labeled with NHS-biotin and then spiked in unlabeled human (HeLa) proteins (1:50, weight) (Fig. 1a). The two-proteome model mimicked the low abundant biotinylated proteins in the unlabeled protein matrix within a biological environment, allowing us to confidently distinguish non-specific bindings (human) from true enrichment (yeast). We first used the two-proteome model to compare biotinylation enrichment at the protein-level and peptide-level using streptavidin magnetic beads (Fig. 1b). As shown in Fig. 1c and Supplementary Fig. S1a, protein-level enrichment yielded three-fold more yeast proteins/peptides compared to peptide-level enrichment. But peptide-level enrichment provided significantly more biotinylated peptides and fewer non-specific bindings.

Enabled by this two-proteome model, we systematically compared commonly used biotinylation enrichment methods using streptavidin (SA), neutravidin (NA), and biotin antibody (BA) coated beads. The binding capacity of each beads type was determined by a dot blot-based beads titration assay (Fig. 1d, 1e)^23^. Proteomics experiments using different beads/protein ratios validated the binding capacity determined by the dot blot assay (Fig. 1f, Supplementary Fig. S1b). The binding capacities at both protein-level and peptide-level were summarized in Fig. 1g. Biotin antibody beads showed the highest binding capacity and enrichment specificity. However, biotin antibody should only be used for peptide-level enrichment due to overwhelming peaks with high charge states (*z* > 5) and elevated LC column pressure from protein-level samples, likely due to antibody contamination from the beads (Supplementary Fig. S1c). Using the optimal beads/protein ratio, we then evaluated the enrichment specificity of the three beads types. NA protein-level enrichment yielded the highest number of total yeast peptides but also the most non-specific bindings (Fig. 1h). BA peptide-level enrichment generated the least total yeast peptides but the most biotinylation sites and the fewest non-specific bindings. SA beads provided the most versatile options with good performance in both protein-level and peptide-level enrichments. When excessive beads were used, SA maintained similar levels of enrichment efficiency and specificity, whereas NA showed declined performances (Fig. 1f). SA beads were also the easiest to handle with rapid pelleting and resuspension. BA beads are incompatible with high detergent concentration and tend to stick to the tube wall or pipette tips, leading to bead loss and variations. Both commercial SA and NA beads offer magnetic bead options for ease of handling and potential automation using magnetic beads processors. It is worth noting that beads from different vendors could have different binding capacities. Optimal beads/protein ratios should be obtained for each beads product and experimental type to ensure sufficient binding efficiency but not excess beads to cause increased non-specific bindings^5^. The performances of different bead types were summarized in radar plots (Fig. 1i) to guide the selection of affinity beads for specific experimental needs.

### Systematic optimizing of biotinylation proteomics sample preparation workflow

**Fig. 2:**
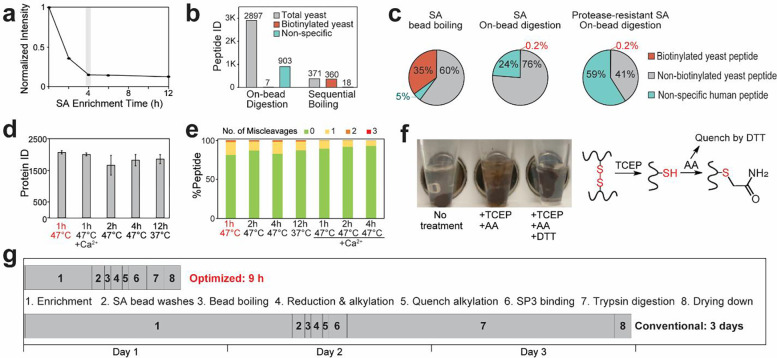
Systematic optimization of the biotinylation proteomics sample preparation workflow. (a) Optimization of SA bead enrichment time by the dot blot assay. Y axis shows the remaining biotinylation signals in the bead-protein mixture supernatant after enrichment. (b) Number of yeast and non-specific human peptides quantified from SA on-bead digestion. Sequential bead boiling after on-bead digestion showed minimal peptide IDs, but majority of peptides were biotinylated. (c) Pie charts showing the percentages of truly enriched yeast peptides and nonspecific bindings from SA bead boiling, SA on-bead digestion, and protease-resistant SA on-bead digestion. Comparison of protein identifications are provided in Supplementary Fig. S2. (d) Comparison of protein IDs under different digestion conditions on SP3 beads (N=3 for each condition). (e) Comparison of peptide miscleavages under different digestion conditions. (f) Picture showing beads performance on a magnetic rack with and without protein reduction (+TCEP), alkylation (+AA), and quenching alkylation reagent (+DTT). The chemical reaction scheme is shown on the right. (g) Schematic comparison of our optimized workflow and conventional workflow.

In order to shorten the complex biotinylation proteomics workflow and reduce variation and protein degradation during sample preparation, we systematically optimized the key parameters involved in each step of the workflow. Biotinylation enrichment is typically done with an overnight incubation. We first optimized the enrichment time, showing complete biotinylation enrichment at 4 hours (Fig. 2a). SA beads from different vendors showed comparable results with 2–4 hours of optimal enrichment time without the need of overnight incubation (Supplementary Fig. S2a). In addition, we found that boiling the leftover SA beads after on-bead digestion can rescue many biotinylated yeast peptides that were not eluted from on-bead digestion (Fig. 2b). SA bead boiling or addition of excess biotin in detergent buffer has been used to competitively elute biotinylated proteins from streptavidin beads for western blotting experiments but can introduce detergent/polymer contaminations for MS-based workflow^24,25^. We found that the recently developed SP3^26^ method could be combined with SA beads boiling to remove contaminants before protein digestion. Indeed, beads boiling followed by SP3 cleanup combined the advantages of both protein-level and peptide-level enrichments, providing excellent total biotinylated proteins and biotinylation sites with minimal non-specific bindings (Fig. 2c, Supplementary Fig. S2b, S2c). We also tested the protease-resistant SA beads which were designed to modify the arginine and lysine residues on SA molecules to block trypsin digestion sites and reduce SA contamination^6^. Despite providing less SA contaminant signals, protease-resistant SA beads showed significantly more non-specific bindings compared to normal SA beads, consistent with a recent report (Fig. 2c, Supplementary Fig. S2c)^27^. This reduced binding specificity is likely due to the negative impact of biotin-SA dissociation constant in modified SA molecules.

Next, we evaluated different protein digestion conditions after beads boiling and protein capture on the SP3 beads. We successfully shortened the protein digestion to only one hour at 47 °C instead of overnight digestion at 37 °C, with slightly improved protein/peptide identifications (Fig. 2d). The addition of calcium ion (Ca^2+^) has previously been shown to enhance trypsin digestion^28^. We found that adding CaCl_2_ slightly reduced miscleavages but did not increase protein/peptide identifications (Fig. 2d, 2e, Supplementary Fig. S2e, S2f). Over 98% of peptides had less than two miscleavages, suggesting acceptable digestion efficiency for all conditions. Since adding CaCl_2_ requires additional desalting step after SP3 digestion, we did not add CaCl_2_ in our optimized digestion workflow. Reduction and alkylation of the protein disulfide bonds are typically conducted before protein digestion. We found that the presence of alkylation reagent, such as iodoacetamide (IAA) or acrylamide (AA), caused SP3 beads aggregation to the tube wall and beads loss during magnetic beads processing (Fig 2f). This is likely because the primary amine group in the alkylation reagent negatively influences the SP3 beads interaction. Therefore, it is crucial to completely quench the alkylation reagent by dithiothreitol (DTT) before adding SP3 beads. As summarized in Fig 2g, we demonstrated a new biotinylation proteomics workflow featuring a 4-hour SA beads enrichment, beads boiling for elution coupled with SP3 clean-up, and an optimized trypsin digestion for 1 hour at 47 °C. The total sample processing time was significantly reduced from 3 days to 9 hours.

### Establishing a fully-automated biotinylation proteomics sample preparation workflow

**Fig. 3:**
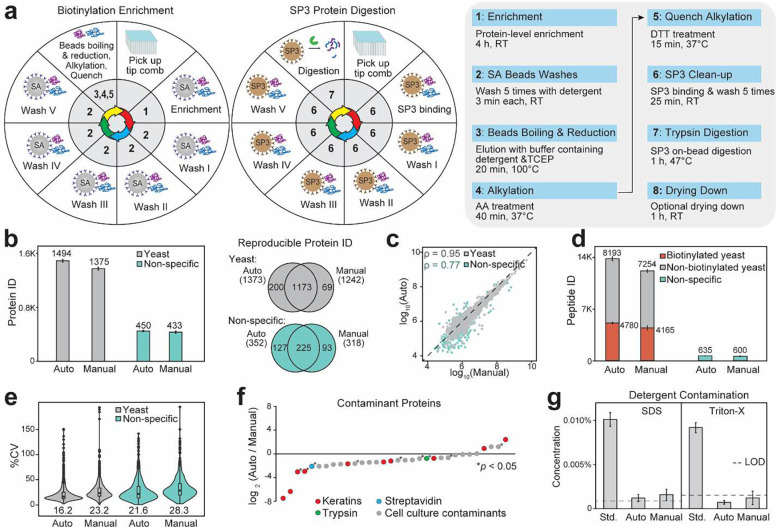
Establishing an automated biotinylation proteomics sample preparation workflow. (a) Schematics of the optimized and automated biotinylation proteomics sample preparation workflow on a 96-well plate KingFisher APEX system with temperature control. Optimization of operating parameters to minimize beads loss is provided in Supplementary Fig S3. (b) Number of proteins quantified from automated vs. manual workflows using the two-proteome model (N=4). Venn diagrams of reproducibly quantified true enrichment yeast and non-specific human proteins from at least 3 replicates are shown on the right. (c) Spearman correlation of protein abundances in the automated vs. manual workflows. (d) Number of yeast and human peptides quantified in the automated vs. manual workflows. (e) Coefficient of variation (CV) distributions of the automated vs. manual workflows. (f) Scatter plot showing contaminant protein ratios of the automated vs. manual workflows. (g) Residue detergent concentrations in LC-MS samples determined by the ContamSPOT assay, compared to 0.01% SDS and Triton standards (N=4). Dashed lines denote ContamSPOT limit of detections (LODs).

With an optimized biotinylation proteomics workflow within a day and the combination of magnetic streptavidin and SP3 beads, we further developed a 96-well plate format automated workflow using the KingFisher APEX platform with temperature control. The schematics of KingFisher plate layout and parameters of key experimental steps were shown in Fig. 3a. We first optimized KingFisher parameters to enhance reproducible beads handling and minimize beads loss (Supplementary Fig. S3). Reducing the enrichment and digestion time from overnight to a few hours significantly minimized beads loss in the automation system. Comparing with the manual proteomics workflow, our automated workflow showed increased protein and peptide IDs with good correlation in quantification (Fig. 3b, 3c, 3d). Over one-third of identified yeast peptides are biotinylated using the new beads boiling elution method for both automated and manual workflows with minimum non-specific bindings (Fig. 3d). Both the optimized 9-hour automated and manual workflows resulted < 4% oxidized peptides in the final proteomics results, significantly lower than the traditional 3-day workflow with ~9% oxidized peptides. In addition, the automated workflow showed reduced quantification variation and contaminant proteins compared to the manual workflow because of reduced human involvement and improved enrichment specificity (Fig. 3e, 3f)^29^. Using our recently developed ContamSPOT assay^30^, we also quantified the trace amount of residue detergents from samples right before LC-MS analysis. Both automated and manual workflows can effectively remove detergents from the original 0.1% SDS and Triton in the lysis buffer, close to or below nanogram levels of detection limits of our ContamSPOT assay (Fig. 3g). Automated workflow showed slightly lower detergent residue compared to the manual workflow. Therefore, our 9-hour biotinylation workflow can be used for both manual and automated sample processing. But automated workflow provided high-throughput capability, increased identification, improved reproducibility, and reduced contamination compared to the manual workflow.

### Applying the optimized and automated workflow to proximity labeling proteomics

**Fig. 4:**
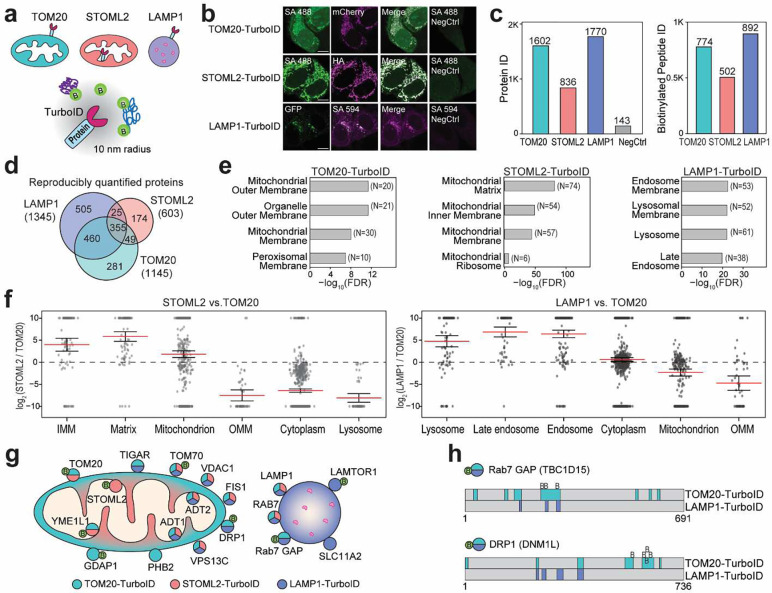
Mitochondrial and lysosomal proximity labeling proteomics. (a) Schematics of TurboID-proximity labeling on the outer mitochondrial membrane (OMM), inner mitochondrial membrane (IMM), and lysosome membrane. (b) Fluorescence microscopy images of TOM20-mCherry-TurboID, STOML2-HA-TurboID, and LAMP1-GFP-TurboID cells after adding biotin, compared to controls without biotin. Compete image panels of control groups are provided in Supplementary Fig. S4. Biotinylated signals were labeled with streptavidin (SA) antibodies. Scale bar denotes 10 μm. (c) Comparison of protein IDs and biotinylated peptide IDs from TOM20-TurboID, STOML2-TurboID, LAMP1-TurboID, and negative control (parental cell line without TurboID). (d) Venn diagram of reproducibly quantified proteins after filtering out proteins in the negative control group. (e) GO enrichment analyses of unique proteins identified in three TurboID groups. (f) Ratio distribution of proteins at known subcellular locations. Lines denote mean ± standard error. (g) Schematic of example key mitochondrial and lysosomal membrane proteins identified in three TurboID-proteomics. (h) Example protein sequence coverages with identified biotinylation sites for two key proteins at the mitochondria-lysosome contact sites.

To demonstrate the applicability of our fully-optimized and automated biotinylation proteomics workflow, we conducted TurboID proximity labeling proteomics in three subcellular locations, TOM20-TurboID on the outer mitochondrial membrane (OMM), Stomatin-like protein 2 (STOML2)-TurboID that is peripherally associated with the inner mitochondrial membrane (IMM) in the matrix, and LAMP1-TurboID on the lysosomal membrane (Fig. 4a). Immunofluorescence imaging confirmed the correct localization of these TurboID probes with significant accumulation of biotinylated proteins at the tubular mitochondria and puncta-like structure of lysosome (Fig. 4b, Supplementary Fig S4a). The optimal beads/protein ratio for TurboID samples was determined by dot blot beads titration assay (Supplementary Fig. S4b). Using our newly developed workflow, we confirmed minimal proteins in the negative control group with limited non-specific bindings (Fig. 4c). TOM20-TurboID and LAMP1-TurboID captured more proteins and biotinylation compared to STOML2-TurboID due to their locations facing the cytosol (Fig. 4c, Supplementary Fig. S4c). Gene ontology (GO) enrichment analysis of the unique proteins reproducibly quantified from three probes confirmed their subcellular locations (Fig. 4d, 4e). Besides expected mitochondrial and lysosomal proteins, cytosolic interactions with mitochondria and lysosomes could also be captured by TurboID-proteomics. Peroxisomal membrane was captured in TOM20-TurboID results, which is known to interact with mitochondrial outer membrane for lipid and reactive oxygen (ROS) metabolism, for example shared fission proteins (DNM1L, FIS1), ROS regulation proteins (PRDX5, SOD1), and peroxisomal membrane proteins (PX11A, PEX14, PX11B, PEX16)^31^. In LAMP1-TurboID proteomics, vesicle trafficking proteins were captured which plays an important role in the maturation of endosomes into lysosomes^32^. Additionally, 95 autophagy-related proteins were captured in LAMP1-TurboID probes, including the vacuolar protein-sorting-associated proteins (VPSs), Ras-related proteins (RABs), and ATG proteins^33^. Comparing STOML2 and TOM20-TurboID provided intra-organelle spatial resolution, where IMM and matrix proteins were highly enriched in STOML2 group and OMM and cytosolic proteins were highly enriched in TOM20 group (Fig 4f, Supplementary Fig. S4d).

Notably, a group of previously reported proteins that regulates the mitochondrial fission event at the mitochondria-lysosome contact sites was enriched in both LAMP1 and TOM20-TurboID proteomics results. These include RAB7 GTPase, DRP1 (a mitochondrial fission factor), RAB GTPase-activating protein (RAB7 GAP) and a mitochondria-resident RAB GAP-binding protein (FIS1) (Fig. 4g)^34^. We also successfully identified biotinylation sites of these proteins (Fig. 4h). These results suggested a successful application of our fully-automated 9-hour workflow to proximity labeling proteomics, capturing the relative proximity information of proteins close to lysosomes or mitochondria, as well as protein transport and communication between these two organelles.

### Dynamic proteome remodeling in mitochondria and lysosomes upon mitochondrial damage

**Fig. 5:**
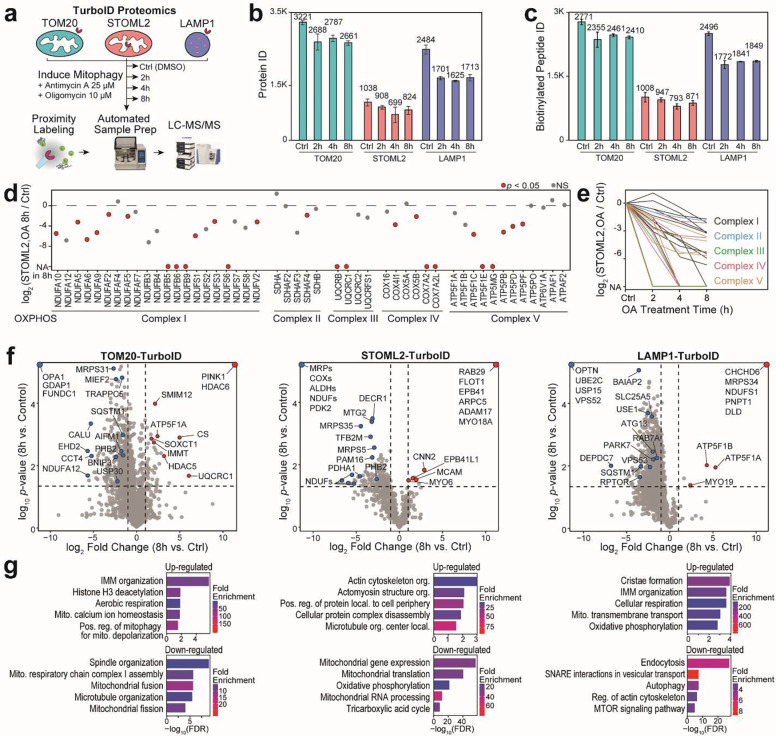
Dynamic protein remodeling during mitochondrial damage. (a) Experimental design to induce mitochondrial damage in HeLa-TurboID cells with different time points of OA treatment and control (DMSO-treated). (b) Number of proteins identified and quantified from TOM20-TurboID, STOML2-TurboID, and LAMP1-TurboID with different drug treatment times (N=3). (c) Number of biotinylated peptide IDs quantified from three TurboID groups. (d) Scatter plot showing OXPHOS protein abundance fold changes (8h vs. control) in STOML2-TurboID proteomics. (e) Time-dependent trends of significantly changed OXPHOS proteins (*p* < 0.05) after OA treatment in STOML2-TurboID proteomics. (f) Volcano plots of TOM20-TurboID, STOML2-TurboID, and LAMP1-TurboID proteomics from 8 h OA treatment vs. control groups. Dashed lines denote *p*-value of 0.05 and fold change of 2. (g) GO enrichment analyses showing enriched biological processes using significantly up-regulated and down-regulated proteins in three TurboID proteomics datasets.

We used above developed automated TurboID proximity labeling platform to probe mitochondrial and lysosomal dynamics during mitochondrial stress. Cells were treated with a combination of two oxidative phosphorylation (OXPHOS) inhibitors, oligomycin and antimycin A (OA), to induce mitochondrial damage^35^. TurboID samples from all three probes were collected at different time points (control, 2, 4, and 8 hours) after OA treatment, allowing us to investigate the time-dependent proteome remodeling across three distinct subcellular locations during mitochondrial stress *in situ* (Fig. 5a). Enabled by our automated workflow, a total of 36 samples were prepared in parallel, resulted in a total of 7369 quantified proteins and 4985 biotinylated peptides (Fig. 5b, 5c). The abundances of bait proteins (TOM20, STOML2, and LAMP1) were not influenced by OA treatment (Supplementary Fig. S5a). OA treatment specifically inhibits OXPHOS complexes, reducing electron transport in mitochondria^36^. We found that majority of OXPHOS subunits were significantly down-regulated after OA treatment in STOML2-TurboID (Fig 5d, 5e).

**Fig. 6:**
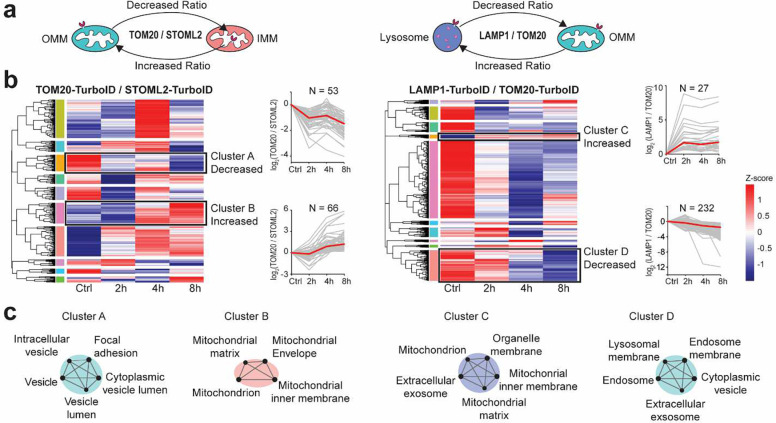
Protein translocation during mitochondrial damage. (a) Schematics to investigate potential protein translocations between outer mitochondrial membrane (OMM), inner mitochondrial membrane (IMM), and lysosome membrane using three TurboID proteomics probes. (b) Hierarchical clustering and heatmaps of average protein abundances in TOM20-TurboID vs. STOML2-TurboID comparison, and LAMP1-TurboID vs. TOM20-TurboID comparison across different drug treatment time points. Only reproducibly quantified proteins from 3 biological replicates are shown here. Protein clusters with consistent increased and decreased trends are shown on the right. (c) GO enrichment analyses showing enriched subcellular locations using consistently increased or decreased proteins from protein clusters.

Mitochondrial damage induced by 8 hours of OA treatment caused dramatic proteome-wide changes in all three probes (Fig. 5f, Fig. 5g). One of the top hits of upregulated proteins in TOM20-TurboID was PINK1, a mitochondrial kinase that plays a critical role in an autophagic degradation of mitochondria, so-called mitophagy (Fig. 5f and Supplementary Fig. S5b)^37^. This result is consistent with a series of previous studies indicating the mitochondrial stress-induced stabilization of PINK1 in the TOM complex, a mitochondrial translocase in the OMM^38,39^. In addition to PINK1, we also observed the up-regulation of HDAC6, a previously reported mitophagy-related factor (Fig. 5f and Supplementary Fig. S5b)^40^. Proteins related to mitochondrial fission/fusion, respiratory chain, and mitochondrial translation were down-regulated in TOM20-TurboID and STOML2-TurboID proteomics, validating impaired mitochondrial function due to OA treatment. IMM proteins were upregulated in both TOM20-TurboID and LAMP1-TurboID probes. Several actin-related cytoskeleton proteins (MYO6, EPB41, CNN2) and endo-lysosome proteins (RAB29 and FLOT1) were significantly up-regulated in STOML2-TurboID. Proteins reproducibly quantified in all time points for each probe were hierarchically clustered based on the changing trend of abundances (Supplementary Fig. S5c, S5d). Interestingly, many proteins enriched in mitophagy, selective autophagy, and lysosome transport were clustered as a recovered trend with decreased abundance after 2 hours of treatment but increased or recovered trend in 4 h and 8 h after the OA treatment. This suggested a recovery response to mitigate mitochondrial damage and important role of vesicle fusion and organization during mitochondrial damage (Supplementary Fig. S5e). To further investigate potential proteome translocation during mitochondrial stress, we compared proteins abundances quantified across different TurboID probes during different time points of OA treatment (Fig. 6a, 6b, 6c). As shown in the hierarchical clustering based on the changing trend of protein ratios, a decreased trend in TOM20/STOML2 TurboID comparison (Cluster A) might indicate that proteins near OMM were translocated to IMM or matrix. These proteins were enriched for cytosolic locations in GO enrichment analysis, possibly suggesting mitochondrial damage during OA treatment. An increased trend (Cluster B) may indicate mitochondrial IMM and matrix proteins translocated to OMM in damaged mitochondria. LAMP1/TOM20 TurboID comparison showed mitochondrial proteins translocating from OMM to lysosome membrane (increased trend in Cluster C) and endosome-lysosome proteins translocating to OMM (decreased trend in Cluster D) during mitochondrial damage. These findings further evidenced the elevated mitochondria-lysosome crosstalk upon mitochondrial damage.

## Discussions

Various strategies are available to enrich biotinylation with pros and cons for each method, but a systematic evaluation of these methods is lacking in the field^8–12^. Here, we addressed this critical gap by designing a two-proteome model to distinguish truly enriched biotinylated proteins from non-specific bindings, enabling comprehensive evaluation of different biotinylation enrichment strategies at the protein-level and peptide-level, as well as different types of enrichment beads (streptavidin, neutravidin, biotin antibody, and protease-resistant streptavidin). We also demonstrated a new strategy with streptavidin beads boiling coupled with SP3 protein digestion to combine the advantages of both protein-level and peptide-level enrichments. We provided practical guideline and suggestions for users to select the most appropriate method for custom experiments. By optimizing the experimental parameters in every step of the workflow, we developed a significantly shortened biotinylation proteomics pipeline within 9 hours instead of the traditional 3-day complex workflow, enabling fully-automated sample preparation in a 96-well plate format. This optimized and automated biotinylation sample preparation workflow achieved comprehensive biotinylation protein and peptide coverages, excellent reproducibility, high-throughput, and minimized non-specific bindings and contaminations.

Mitochondria and lysosomes are important membrane-bound organelles in the cell that are responsible for ATP production and trash-disposal, respectively^41,42^. Enabled by our newly established automated biotinylation proteomics workflow, we successfully characterized mitochondrial and lysosomal microenvironment with sub-organelle spatial resolution in both healthy state and under mitochondrial damage. Mitochondria and lysosomes frequently interact with each other for mitochondrial quality control (MQC), metabolism, and signaling pathways^43^. Many of these dynamic activities and transient interactions cannot be captured with organelle isolation or immunoprecipitation and are mainly studied by microscopy techniques, one interaction at a time^44,45^. Recent advances in electron and super-resolution microscopy techniques also enabled the visualization of mitochondria-lysosomes contact sites with an average distance of 10 nm in mammalian cells^34,46^ This indicated that proximity labeling with a 10 nm labeling radius could be an alternative tool to pinpoint mitochondria-lysosome contact sites and provide precise contact sites information with high throughput capability. Indeed, our mitochondrial and lysosomal proximity labeling proteomics identified and quantified a group of recently discovered proteins at the mitochondria-lysosome contact sites, RAB7, DRP1, RAB7 GAP (TBC1D15), and FIS1, with precise amino acid residues of biotinylation sites in these proteins. Wong et al. ^34,47^ showed that the lysosomal protein RAB7 promotes the contact formation between lysosomes and mitochondria and regulates mitochondrial fission event. These studies also indicate that DRP1, the GTPase dynamin-related protein that polymerizes and encircles mitochondria for scission^48,49^, accumulates at the mitochondria fission site marked by the lysosome. Conversely, to release the mitochondria-lysosome contact, the mitochondrial protein FIS1 recruits the RAB7 GTPase-activating protein (TBC1D15) to drive RAB7 GTP hydrolysis^34^. To our knowledge, this is the first successful example of unbiased proteomics that captured proteins at the mitochondria-lysosome contact.

Besides the direct interaction between mitochondria and lysosome, mitochondria establish inter-organelle interactions through membrane signaling and tethering molecules that coordinate other MQC pathways, such as mitophagy and MDVs. When mitochondria are damaged, PINK1 stabilizes on the outer mitochondrial membrane and recruits Parkin from the cytosol to mitochondria, triggering the selective autophagic removal of the damaged mitochondria (i.e., mitophagy)^37^. Consistent with these observations, our TOM20-TurboID proteomics successfully captured the mitochondrial stress-dependent PINK1 accumulation on the OMM^50,51^. PINK1 is not detected under steady state conditions as PINK1 is rapidly imported into mitochondria, cleaved, and released back into cytoplasm for proteasomal degradation under the basal condition^52^. In addition to PINK1, HDAC6 was also identified as one of the top hits of our TOM20-TurboID proteomics after OA treatment. Intriguingly, a previous study reported that HDAC6 is a positive regulator of mitophagy that is recruited to mitochondria in Parkin-dependent manner^40^. Conversely, USP30, a mitochondrial deubiquitinase that negatively regulates PINK1/Parkin-mediated mitophagy^53^, was significantly down-regulated in TOM20-TurboID proteomics comparing 8-hour OA treatment to control. Comparing protein ratios across different TurboID probes at different time points of OA treatment could indicate potential dynamic translocation of proteins within or between organelles. For example, with the progression of OA treatment, we observed increased mitochondrial matrix proteins translocated near OMM in TOM20-TurboID probe (e.g., NDUFA12, NDUFS1, NDUFA9, ATP5PD, TIMM50) and increased cytosolic proteins near IMM in the STOML2 probe (e.g., DDX3X, GAPDHS, SEC16A, CORO1C, PEX5). As mitochondrial membrane potential is essential for the import of the majority of proteins in the matrix, these observations may indicate the mitochondrial import arrest of mitochondrial proteins. Remarkably, we also observed the possibility of the elevated mitochondria-lysosome crosstalk during the progress of mitochondrial damage, with increased mitochondrial proteins near lysosome in LAMP1-TurboID (e.g., ATP5F1A, ATP5F1B, COX5B, SDHA, TRAP1, HSPA9), and increased lysosomal proteins near mitochondria in TOM20-TurboID (e.g., TFEB, RB27A, SQSTM1, RHEB, LAMTOR1, VAMP8). Although our HeLa-TurboID cells do not express Parkin, these observations may provide new mechanistic insights into the spatial and temporal regulation of mitophagy-regulatory factors surrounding mitochondria during mitochondrial stress^57^.

In summary, we have developed a two-proteome model to benchmark biotinylation proteomics and a fully-automated 9-hour biotinylation proteomics workflow that can be broadly used to study protein interaction and organelle dynamics. Applying this workflow to proximity labeling allowed us to track dynamic proteome changes at multiple time points and across two distinct but functionally associated organelles, mitochondria and lysosome, during mitochondrial damage. While we sought to provide a comprehensive method evaluation and application of our new workflow, it is important to acknowledge the limitations of this study. For benchmarking and method comparisons, we mainly focused on the methods that were well-documented in the literature and worked well in our hands. It was not an exhaustive evaluation of all possible methods for biotinylation enrichment. For automating the biotinylation proteomics workflow, we mainly optimized the KingFisher APEX 96-well plate magnetic processor. Other automated platforms such as liquid handlers can also be used^13,14^. For the application of our optimized and automated biotinylation proteomic workflow, we have successfully demonstrated TurboID proximity labeling proteomics and its application in understanding mitochondria-lysosome interactions. Future follow-up studies are needed to validate the changes of individual proteins to further understand the detailed molecular mechanisms in MQC pathway and the dynamic mitochondrial-lysosome crosstalk in healthy and diseased states.

## Methods

### Cell culture and TurboID cell lines

HeLa cells were maintained in DMEM/F12 HEPES medium (Gibco) supplemented with 10% fetal bovine serum (Gibco) and 1x penicillin-streptomycin solution at 37 °C and 5% CO_2_. Three TurboID HeLa cell lines were generated by stably expressing TurboID onto specific sub-organelle locations, including mitochondrial outer membrane facing cytosol (TOM20-mCherry-TurboID), mitochondrial inner membrane facing mitochondrial matrix (STOML2-HA-TurboID), and lysosomal membrane facing cytosol (LAMP1-GFP-TurboID) using lentivirus transfection and the well-established TurboID method from the Ting group^8,22,54^. Parental and TurboID cells were cultured in 10 cm dishes until 80% confluence. Cells were incubated with 50 μM biotin (Cayman) at 37 °C for 30 min to activate proximity labeling, gently washed with phosphate-buffered saline (PBS) for three times, and pelleted by Trypsin-EDTA. To induce mitochondrial damage, cells were treated with a combination of 10 μM oligomycin and 25 μM antimycin A (OA) and harvested at different time points (control, 2 h, 4 h, 8 h) after treatment. Biotin was added for a shorter period with higher concentration (15 min, 500 μM) in the drug treatment experiment to provide better temporal resolution for different time points.

### Two-proteome model with biotinylated yeast and unlabeled HeLa proteins

To create the two-proteome model, yeast proteins were labeled with amine-reactive NHS-biotin (APExBIO) and spiked in unlabeled HeLa protein lysate with a 1:50 ratio (yeast : human, w/w) with the following steps: HeLa cell pellet from the parental cell line was lysed with a buffer containing 0.1% SDS, 0.1% Triton-X, 5 mM ammonium bicarbonate (AmBc), and 100 mM sodium chloride (NaCl). HeLa cell lysate was sonicated for 15 min using a QSonica Q700 sonicator with 1 min on and 30 s off cycles in ice slurry, followed by 15 min centrifugation at 12,700 rpm at 4 °C. Protein concentration of the supernatant was determined using a detergent-compatible colorimetric protein assay (DCA, Bio-Rad). Yeast protein extract (Baker’s yeast) was prepared with the same step as the HeLa cell lysate. NHS-biotin (100 mM in DMSO) was mixed with yeast protein with a 200-fold molar ratio and vortexed for 30 min in a ThermoMixer at 25 °C. The labeling reaction was quenched with 10% hydroxylamine for 15 min. Cold acetone (−30 °C, 4-fold volume) was added to precipitate biotinylated yeast proteins. The protein pellet was centrifuged and washed with cold acetone three times to remove excess NHS-biotin, followed by resuspension with a buffer containing 1 M urea, 50 mM AmBc, and 150 mM NaCl. A mixture of biotinylated yeast proteins and human HeLa proteins (1:50, w/w) was stored in −80 °C until use.

### Beads titration using a dot blot assay

The optimal beads/protein (μl/μg) ratio was determined by beads titration assay as described previously^5,23^. Briefly, 20 μg of biotinylated protein or peptide was added to each 0.5 mL tube that containing a series volume of enrichment beads slurry. Different beads used here include streptavidin magnetic beads (Cytiva), neutravidin agarose beads (Thermo Scientific), anti-biotin antibody agarose beads (ImmuneChem), protease-resistant streptavidin magnetic beads (ReSyn), and High-bind streptavidin magnetic beads (Agilent). After overnight rotation at 4 °C, 2 μL of the supernatant from each tube was spotted on a nitrocellulose membrane. The dried membrane was incubated with blocking buffer that contains 5% (w/w) bovine serum albumin (BSA, Fisher Bioreagents), 0.05% Tween 20, 20 mM Tris-HCl, 150 mM NaCl for 1 h, followed by overnight incubation with Streptavidin Alexa Fluor^™^ 647 conjugate (1:1000 in blocking buffer). The membrane was washed five times with a buffer containing 0.05% Tween 20, 20 mM Tris-HCl, 150 mM NaCl. The fluorescent signal was measured using a ChemiDoc MP imaging system (Bio-Rad) with a filter of 647 nm. The signal intensity was quantified using ImageJ^55^.

### Biotinylation enrichment for proteomics at the protein-level and peptide-level

SA, NA, and BA beads were compared for both protein-level and peptide-level enrichment of biotinylation. For protein-level enrichment, beads were mixed with protein lysate at optimal beads/protein ratio and rotated overnight at 4 °C. Beads were washed two times with beads wash buffer containing 0.1% SDS, 0.1% Triton-X, 5 mM AmBc, and 100 mM NaCl, one time with PBS, and one time with water. For SA beads, captured proteins were eluted by on-bead digestion as described previously^5,8^. Briefly, SA beads were treated with 5 mM tris(2-carboxyethyl)phosphine (TCEP) for 40 min and 15 mM acrylamide (AA) for 30 min, followed by 15 mM dithiothreitol (DTT) for 15 min on a ThermoMixer. Trypsin/Lys-C (Promega) was added to the SA beads for a 16 h digestion. The supernatant was collected and heated to 99 °C for 5 min to deactivate trypsin and quench the reaction. For NA and BA beads, proteins were eluted by a buffer containing 100 μL of 0.2% trifluoroacetic acid (TFA) and 0.1% formic acid (FA) in water for 15 min at 37 °C. This elution was repeated once, and supernatant was combined. The eluted protein from NA and BA beads was reduced, alkylated, digested, and quenched with the same steps as the SA beads, except in-solution. All digested peptide samples were stored at −30 °C until further clean-up.

For peptide-level enrichment, protein lysate was first digested by Trypsin/Lys-C (1:30 weight ratio to protein) and quenched by heating the sample tube to 99°C for 5 min. Beads were mixed with protein lysate at optimal beads/protein ratio and rotated overnight at 4 °C, followed by the same beads washing steps as the protein-level enrichment. For SA beads, biotinylated peptides were eluted by beads boiling in a buffer containing 1% SDS, 20 mM TCEP, 5 mM AmBc, and 2 mM biotin at 100 °C for 15 min. This elution was repeated once, and supernatant was combined. For NA and BA beads, peptide-level and protein-level elution used the same steps and buffer. Peptides were cleaned using the SP2 method^56^. Briefly, 10 μL of mixed Sera-Mag hydrophilic and hydrophobic magnetic particles (Cytiva) were added to the peptides. Acetonitrile (ACN) was added to a final concentration of 95%. The sample was incubated at 25 °C for 15 min on a ThermoMixer. After removing the supernatant, the beads were washed five times with 95% ACN. Peptides were eluted twice with 100 μL LC-MS grade water at 25 °C for 10 min. Peptides were dried and stored at −30 °C.

### Automated and optimized sample preparation for biotinylation proteomics

KingFisher Apex system (Thermo Fisher) was used for fully automated biotinylation proteomics sample preparation in a 96-well plate format. Protein lysate and SA beads were added to a 96-well plate. Gentle mixing was applied for enrichment at 4 °C for 4 h. SA beads were collected by magnet and washed five times at 4 °C with a buffer containing 1% SDS, 1% Triton-X, 50 mM AmBc, and 150 mM NaCl. Enriched proteins were eluted by boiling at 100 °C for 20 min in a buffer containing 1% SDS, 20 mM TCEP, 5 mM AmBc, and 2 mM biotin. Eluted proteins were alkylated with 15 mM AA for 40 min at 37 °C, and excessive AA was removed by treating with 15 mM DTT for 15 min at 37 °C. Proteins were then cleaned and digested on the automated KingFisher platform using the SP3 method^26^. Briefly, ACN was added to each sample to a final concentration of 80%, followed by an addition of 10 μL SP3 beads (Cytiva) for a 10 min incubation to induce protein binding. The beads were washed three times with 95% ACN and then two times with 70% ethanol. The beads were then released into a 100 μL 50 mM AmBc buffer. Trypsin/Lys-C was added to the beads for protein digestion at 47 °C for 1 h with gentle mixing. Beads were collected and washed again with 100 μL LC-MS grade water to combine with the supernatant. The supernatant samples in a 96-well plate were dried under SpeedVac and stored at −30 °C.

### Contaminant spot check and removal assay (ContamSPOT)

Peptide samples were tested for potential detergent contamination before LC-MS using our newly developed ContamSPOT assay as described previously^30^. To test SDS residue, one microliter of the sample was mixed with 1 μL of 0.1% o-toluidine blue (Carolina Biological), and 3 μL of ethyl acetate (Fisher Scientific) in a 0.2 mL tube, vortexed, and briefly centrifuged. Two microliters of the top ethyl acetate layer were spotted on a thin layer chromatography (TLC) plate (Sorbtech Silica). A photo of the spot was taken for quantification by ImageJ software or directly visualized by the naked eye for the presence (blue residue) or absence of contamination. To test Triton residue, one microliter of the sample was mixed with 2 μL of 1X Dye A43 and 1 μL of 1X Reagent 2 from the ProFoldin Detergent Assay Kit (DAK 1000) for 5 min then spotted on a TLC plate for fluorometric imaging under 632 nm in the ChemiDoc MP imaging system. For accurate quantification, calibration curves were established using a series concentration of SDS and Triton standards. If ContamSPOT tested positive, ethyl acetate liquid-liquid extraction can be conducted to remove trace detergents as described previously^30^. Briefly, a 10-fold volume of water saturated ethyl acetate was added to the sample to vortex and centrifuge for 30 s each. After 3 ethyl acetate washes, the top ethyl acetate layer was carefully removed, and samples were dried for subsequent LC-MS/MS analysis.

### LC-MS/MS analysis

Peptide samples were resuspended in 2% ACN, 0.1% FA in LC-MS grade water and clarified by centrifugation before adding to LC vials. LC-MS/MS analyses were conducted on a Dionex Ultimate 3000 RSLCnano system coupled with a Thermo Scientific Q-Exactive HFX or Fusion Lumos mass spectrometer. The mobile phase A was 0.1% FA in water, and mobile phase B was 0.1% FA in ACN. Peptide were separated on an Easy-spray PepMap C18 column (3 μm, 100 Å, 75 μm × 15 cm) with a 2-hour LC gradient and 55 °C column temperature. The flow rate was 0.3 μL/min. To achieve better separation for TurboID samples, a longer column (2 μm, 100 Å, 75 μm × 50 cm) was used with a 3-hour LC gradient and 55 °C column temperature. The flow rate was 0.25 μL/min. The quadrupole mass filtering was set from *m/z* 400 to 1500 with a resolving power of 60,000 (at *m/z* 200 FWHM). A top 25 data-dependent acquisition was used for MS/MS with a resolving power of 15,000. Parent masses were isolated with a *m/z* 1.4 window and fragmented with higher-energy collision dissociation (HCD). The normalized collision energy was 30% and the dynamic exclusion time was 22.5 seconds. The maximum injection times were 30 ms for MS and 35 ms for MS/MS. The automatic gain control (AGC) was 1 × 10^6^ for MS and 1 × 10^4^ for MS/MS.

### Proteomics data analysis

LC-MS/MS raw files were analyzed using the Thermo Fisher Proteome Discoverer software (2.4.1.15) with the SequestHT search engine. Swiss-Prot *Homo sapiens* and *S. cerevisiae* databases were used for human and yeast protein identifications, respectively. A cell-culture specific contaminant FASTA library, established in our laboratory, was used to mark and remove contaminant proteins (available to download at https://github.com/HaoGroup-ProtContLib)^57^. False discovery rate cutoff was set at 1% for protein and peptide spectral match identifications. Trypsin was used as the enzyme with four maximum miscleavages. Cysteine alkylation was included as a fixed modification. Methionine oxidation and acetylation of protein N-terminus were included as variable modifications. Biotinylation (+226.0776 Da) at lysine residue was added as a variable modification. Protein GO enrichment analysis was conducted using ShinyGo^58^ online software.

### Statistical analysis

Statistical analysis was conducted with two-tailed unpaired Student’s t-test. Hierarchical clustering was conducted using the pheatmap function in R studio. The number of replicates was provided in figure legends. Error bars in figures denote standard deviation from at least 3 replicates. Results of statistical analysis are also provided in supplementary data and source data.

### Immunocytochemistry

Cells were seeded into Lab-Tek Chambered Coverglass with 4 wells (Thermo Fisher Scientific). Cells were rinsed in PBS and fixed with PBS containing 4% formaldehyde for 15 min at room temperature (RT). Cells were permeabilized with PBS containing 0.1% Triton X-100 for 10 min at RT. Blocking was performed using PBS containing 2% BSA for 30–60 min at RT. For immunostaining, cells were incubated with primary or secondary antibodies diluted in PBS containing 2% BSA for overnight at 4 °C or about 2 h at RT, respectively. Cells were imaged using a 63 × /1.4 NA oil immersion objective on Leica SP8 LIGHTNING Confocal Microscope (Leica). Following antibodies and fluorescent probes were utilized; HA (Cell Signaling, 3724), Streptavidin, Alexa Fluor^™^ 594 conjugate (Invitrogen, S11227), Streptavidin, Alexa Fluor^™^ 488 conjugate (Invitrogen, S11223), Donkey anti-Rabbit IgG Alexa Fluor^™^ Plus 555 (Invitrogen, A32794).

## Supplementary Material

Supplement 1

## Data Availability

The MS proteomics data have been deposited to the ProteomeXchange Consortium via the MassIVE repository (ftp://MSV000094795@massive.ucsd.edu). Other data and detailed proteomics results associated with this study are available in Supplementary Data and Source Data.
