# Supplementary material for: Benchmarking and Automating the Biotinylation Proteomics Workflow
Source: Res Sq. 2024 Jul 3:rs.3.rs-4590410. Preprint. [Version 1] doi: 10.21203/rs.3.rs-4590410/v1 (PMC11247940; doi:10.21203/rs.3.rs-4590410/v1)
Supplement: Supplement 1 [file NIHPPrs4590410v1-supplement-1.pdf]

Supplementary information

- Supplementary Figure S1: Evaluating different methods to enrich biotinylation.
- Supplementary Figure S2: Optimizing streptavidin beads enrichment and SP3 digestion conditions.
- Supplementary Figure S3: Optimizing mixing parameters and enrichment times on an automated 96-well plate KingFisher APEX system.
- Supplementary Figure S4: Mitochondrial and lysosomal proximity labeling proteomics
- Supplementary Figure S5: Dynamic protein remodeling during mitochondrial damage.
- Supplementary Data S1: TOM20-TurboID, STOML2-TurboID, and LAMP1-TurboID proteomics results in healthy cells.

- Supplementary Data S2: TurboID proteomics results during OA treatment
- Supplementary Data S3: TurboID protein clusters showing protein abundance and potential translocation during mitochondrial damage
- Source Data.

Supplementary Figures and Figure Legends

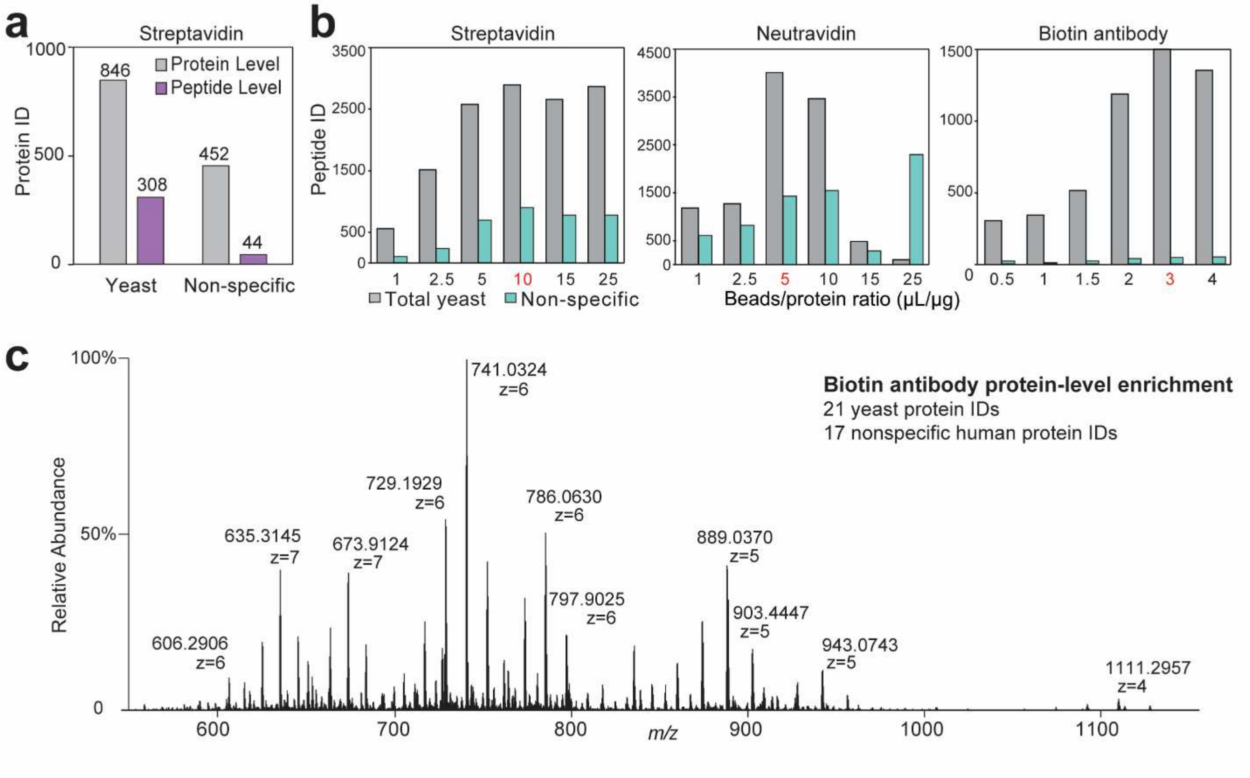

Supplementary Fig. S1: Evaluating different methods to enrich biotinylation. (a) Number of proteins quantified in protein-level and peptide-level biotinylation enrichments using streptavidin magnetic beads. Protein-level enrichment provided more true enrichment (yeast proteins) but also more nonspecific bindings (human) compared to peptide-level enrichment. (b) Number of peptides quantified from SA, NA, and BA enrichment with different beads/protein ratios. Optimal ratio was marked in red. True enrichment of yeast proteins and non-specific human proteins are labeled in grey and cyan, respectively. (c) Example MS spectrum from biotin antibody protein-level enrichment samples showing limited protein identifications and highly charged peaks from antibody contamination. Biotin antibody should only be used for peptide-level enrichment and should not be used for protein-level enrichment.

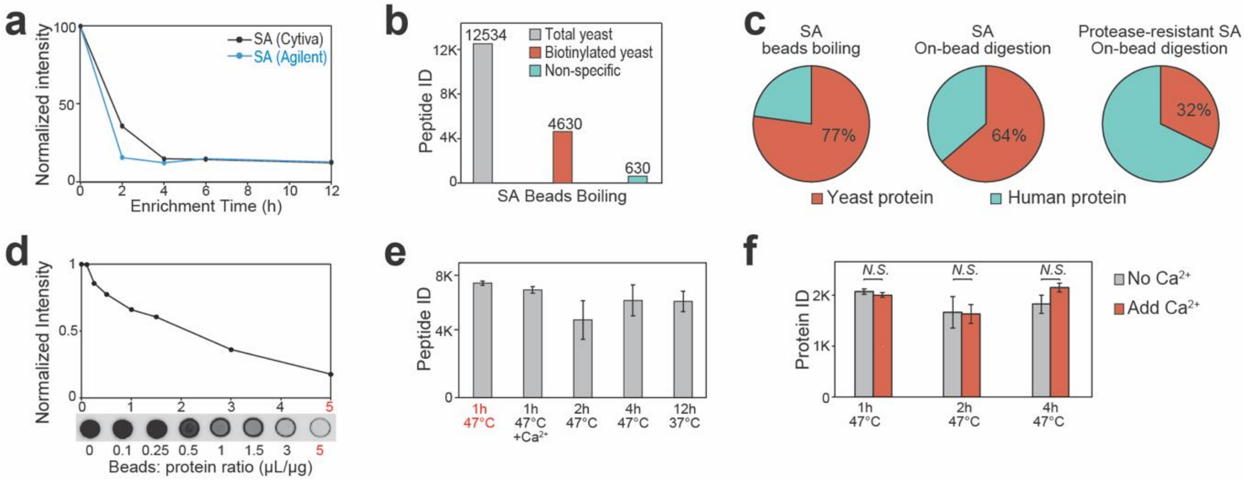

Supplementary Fig. S2: Optimizing streptavidin beads enrichment and SP3 digestion conditions. (a) Comparison of different biotinylation enrichment times using streptavidin beads from different vendors. The y-axis shows the remaining biotinylation signals in the bead-protein mixture supernatant after enrichment. (b) Number of peptides quantified from streptavidin bead boiling followed by SP3 cleanup. True enrichment of yeast peptides and non-specific human peptides are labeled in different colors. (c) Pie charts showing the percentages of yeast proteins and nonspecific-binding human proteins from SA bead boiling, SA on-bead digestion, and protease-resistant SA on-bead digestion. Normal SA beads are from Cytiva, and protease-resistant beads are from ReSyn. (d) Binding capacity of protease-resistant streptavidin beads using dot blot assay. The optimal ratio is marked in red. (e) Comparison of peptide IDs under different digestion conditions on SP3 beads (N=3). (f) Comparison of protein IDs with and without adding calcium chloride under different SP3 digestion conditions (N=3).

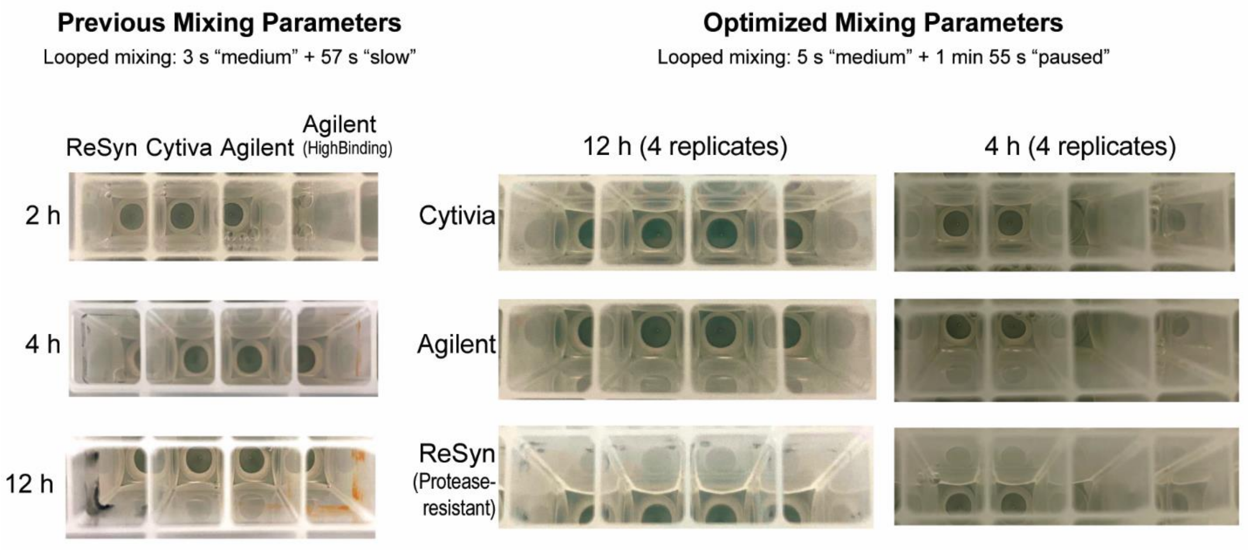

Supplementary Fig. S3: Optimizing mixing parameters and enrichment times on an automated 96-well plate KingFisher APEX system. Pictures showing the extent of beads loss on the 96-well plate walls after beads enrichment. Streptavidin magnetic beads from different vendors (Cytivia, Agilent, and ReSyn) were used. Longer enrichment time caused more beads loss on the 96-well plate walls. No beads loss was observed with the optimized mixing parameters after 4-hour biotinylation enrichment at room temperature.

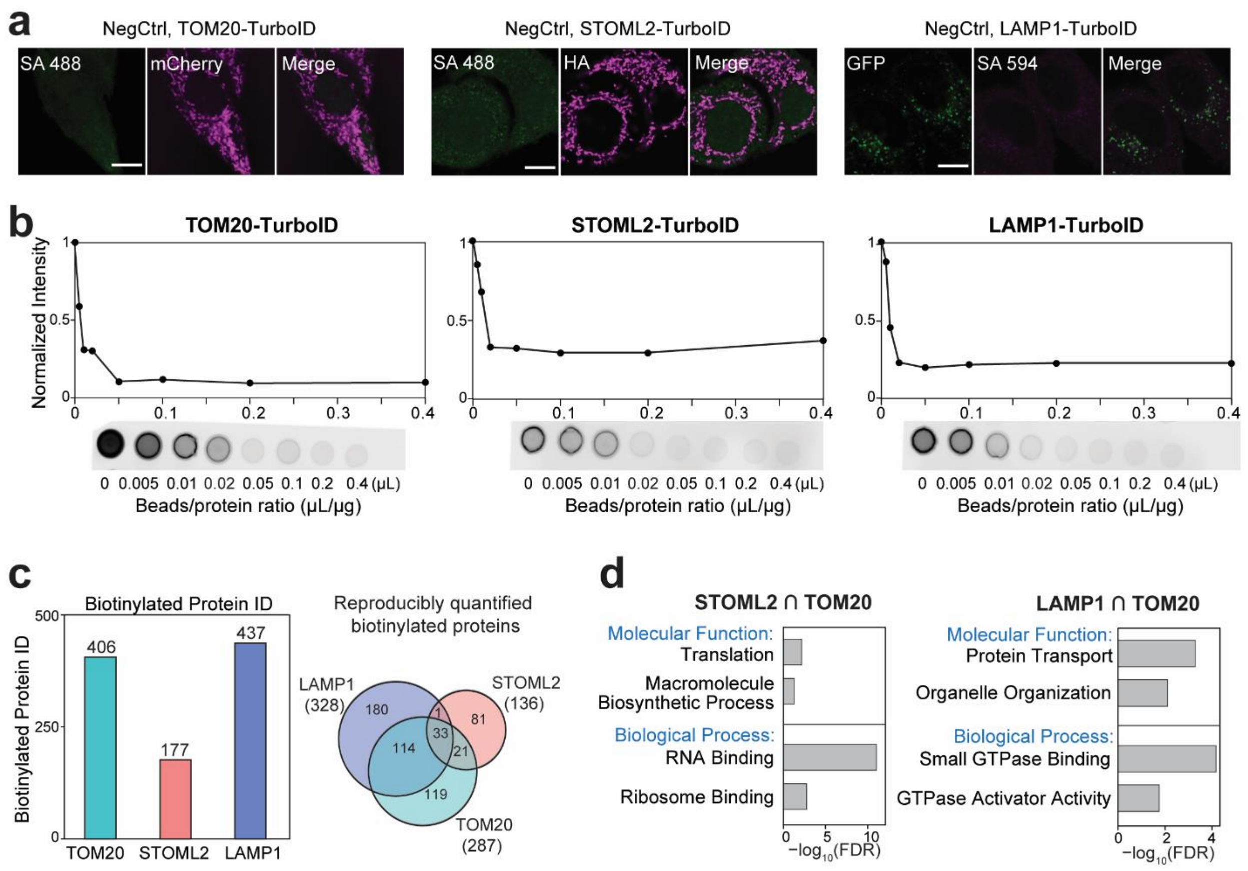

Supplementary Fig. S4: Applying the automated workflow to mitochondrial and lysosomal TurboID proximity labeling proteomics. (a) Fluorescence microscopy images of negative control cells without adding biotin substrate in TOM20-mCherry-TurboID, STOML2-HA-TurboID, and LAMP1-GFP-TurboID cells. Biotinylated signals were labeled with streptavidin (SA) antibodies. Scale bar denotes 10 µm. (b) Beads titration using dot blot assay to determine optimal beads/protein ratios for TurboID cell samples. (c) Number of biotinylated proteins identified from TOM20-TurboID, STOML2-TurboID, and LAMP1-TurboID cells. (d) GO enrichment analyses using proteins reproducibly quantified in both STOML2-TurboID & TOM20-TurboID groups (left) and LAMP1-TurboID & TOM 20-TurboID groups (right).

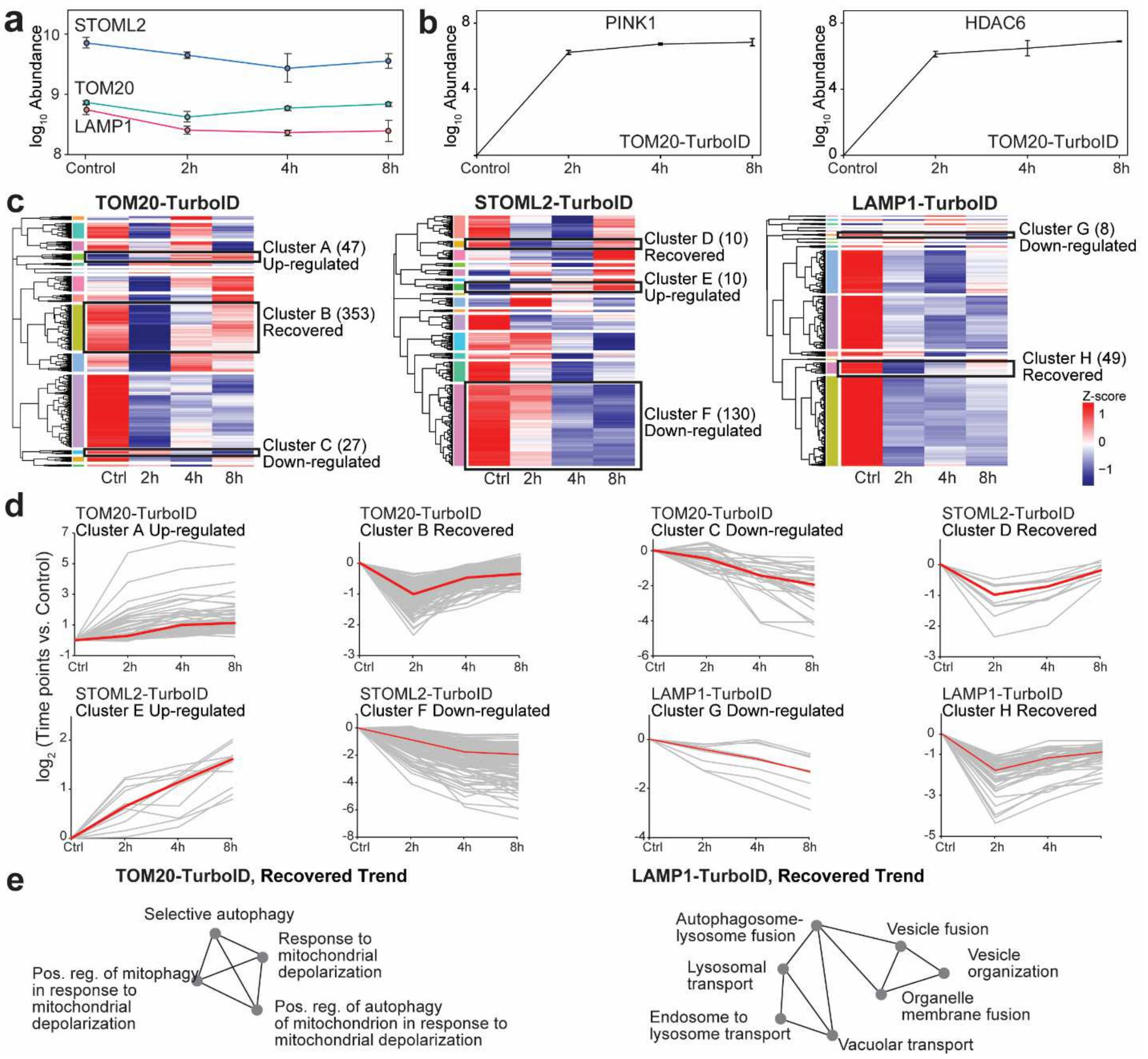

Supplementary Fig. S5: Mitochondrial and lysosomal proximity labeling proteomics during mitochondrial damage. (a) Bait protein abundances across different OA drug treatment time points. (b) Up-regulated PINK1 and HDAC6 in TOM20-TurboID indicated their accumulation to the outer mitochondrial membrane during mitochondrial damage. (c) Hierarchical clustering and heatmaps of average protein abundances in TOM20-TurboID, STOML2-TurboID, and LAMP1-TurboID proteomics in different drug treatment time points. Only reproducibly quantified proteins from 3 biological replicates are shown here. (d) Protein clusters with consistent up-regulation, down-regulation, and recovered trends across different drug treatment time points. (e) GO enrichment analysis showing enriched biological processes from proteins with recovered trends in TOM20-TurboID and LAMP1-TurboID protein clustering.
